# The Danger of Flannelette

**Published:** 1910-10-29

**Authors:** A. F. Edwards

**Affiliations:** Victoria University of Manchester


### Editor's Letter-Box.

### THE DANGER OF FLANNELETTE.

*To the Editor of* The Hospital.

Sir,—The publicity which the Press has given to the Countess of Ancaster's appeal to needlework guilds with reference to the danger of flannelette is another sign of the widespread interest now being taken in the cause of the children. With the coming of the cold and dark days of winter the charitably disposed and the many commendable societies which at this time make an extra effort to relieve, as far as possible, the suffering brought to their notice will be considering the best and readiest means of doing this.

One of the most welcome forms which this kindness takes is that of supplying seasonable clothing to the aged and feeble, and to young children in particular; and a favourite article for such clothing is flannelette—a material which is warm, comfortable, cheap, and extremely dangerous. It is common knowledge that a Royal Commission has recently been considering the matter of the enormous number of deaths through burns caused by the wearing of flannelette which take place annually, and more particularly in the winter months. It was admitted that in three months of this period last year 176 such fatal accidents had been fully investigated, the great majority being quite young children. But it may not be so well known that after commenting upon this the Committee, in their report, say: "A process which renders the stuff comparatively uninflammable, notwithstanding repeated washings, and which at the same time does not enhance the price prohibitively, is what is required. Hitherto there appears to be only one process which at all complies with these conditions—namely, Dr. Perkin's process 'Nonflam.'"

The National Society for the Prevention of Cruelty to Children have also recently issued a pamphlet dealing with the flannelette danger, and recommending the use of the same material for the wear of children; and the British Fire Prevention Committee, after having made a series of over 450 exhaustive tests during the last year, have printed a booklet also recommending this as the only safe flannelette, being practically equal to flannel in this respect.

Victoria University of Manchester: October 20.

Yours faithfully,

A. F. Edwards.

